# Supplementary material for: Microdroplets initiate organic-inorganic interactions and mass transfer in thermal hydrous geosystems
Source: Nat Commun. 2024 Jun 11;15:4960. doi: 10.1038/s41467-024-49293-y (PMC11167059; doi:10.1038/s41467-024-49293-y)
Supplement: Supplementary file 1 — Supplementary Information [file 41467_2024_49293_MOESM1_ESM.pdf]

Supplementary information

Microdroplets initiate organic-inorganic interactions and mass transfer in thermal hydrous geosystems

Yuan et al.

Supplementary Figures

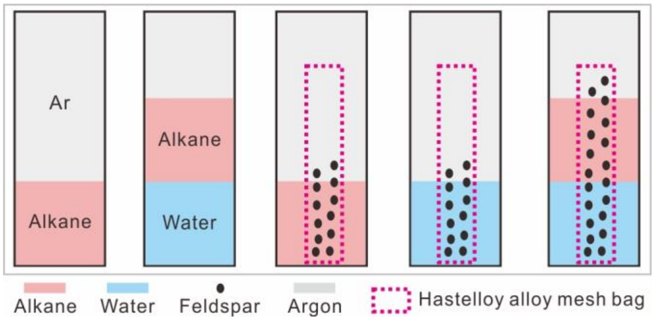

Supplementary Fig. 1. Physical configuration of alkane, water and feldspar grains in the isotope-tagged thermal experiments. Samples of water, *n*-eicosane and feldspar grains in mesh bags were placed into Hastelloy reactors with different combination of these species (Supplementary Table 1). After loading the samples, the air in the reactors was removed with argon, and the reactors were then sealed in the presence of argon. Mesh bags were used to ensure contact between the minerals and water in the lower part of the reactors and the alkane in the upper part.

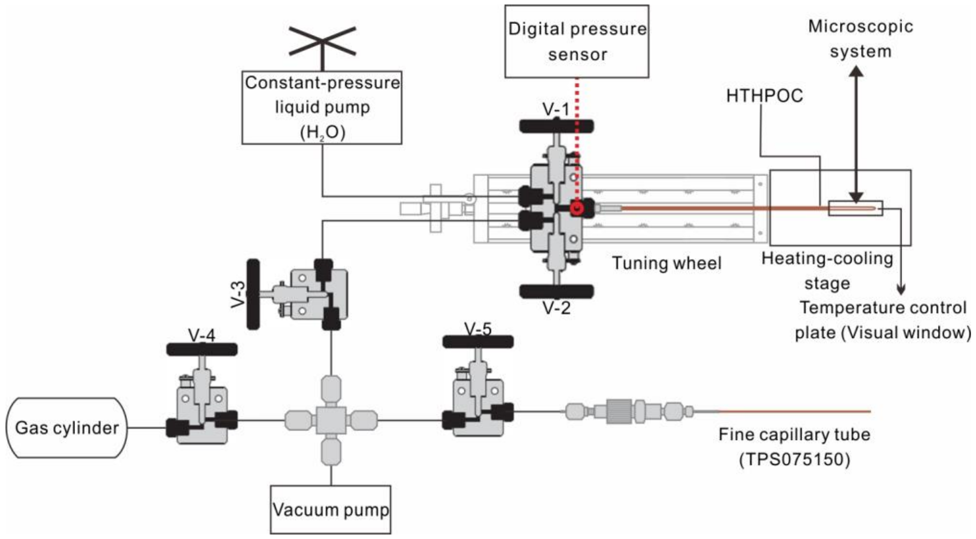

Supplementary Fig. 2. Schematic diagram of the transparent silica capillary thermal experimental system[1]. The system is composed of a HTHP silica capillary tube optical cell (HTHPOC), a microscopic system with camera and video recording module, a heating-cooling stage, a tuning wheel, a constant pressure liquid pump, a digital pressure sensor, a vacuum pump system, and a gas cylinder. V-1, V-2, V-3, V-4, V-5 represent valves for different use. The HTHPOC is used to create oil-water system with interface, the heating-cooling stage is used to increase experiment temperature, the constant pressure liquid pump and digital pressure sensor are used to increase and test pressure in the HTHPOC, the microscopic system with camera and video recording module is used to observe and record the phenomena in the HTHPOC, the tuning wheel is used to finely adjust the position of HTHOPC. (re-use permission has been obtained from Springer Nature).

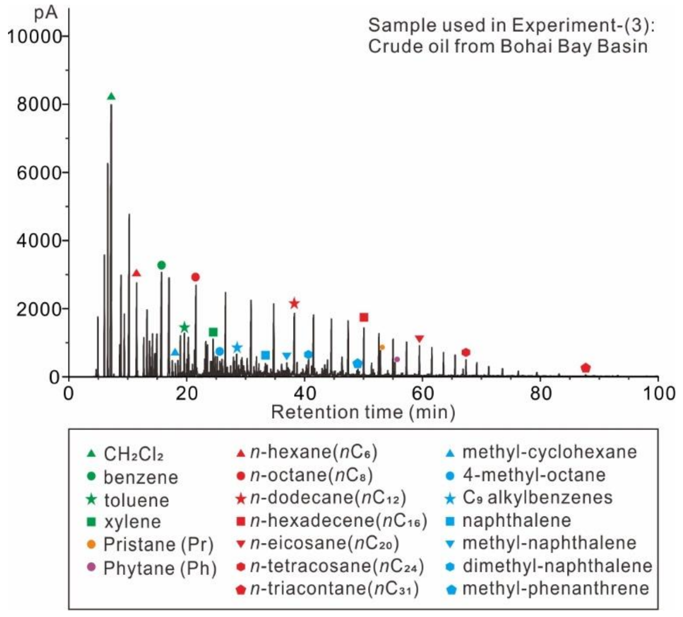

Supplementary Fig. 3. Gas chromatograms of the crude oil from the Bohai Bay Basin, East China used in the visual experiments. *n*-alkanes (nC_6-nC_{26}) dominated the main compositions of the crude oil. Additionally, mono-bicyclic aromatic hydrocarbons (benzene, toluene, xylene, C_9 alkylbenzenes, naphthalene, methyl-naphthalene, dimethyl-naphthalene), polycyclic aromatic hydrocarbons (methyl-phenanthrene), and biological precursors including pristane (Pr) and phytane (Ph) were clearly identifiable. The crude oil was employed in the in-situ visual experiments to verify that that water microdroplets can form in crude oil near the oil-water interface at elevated temperatures.

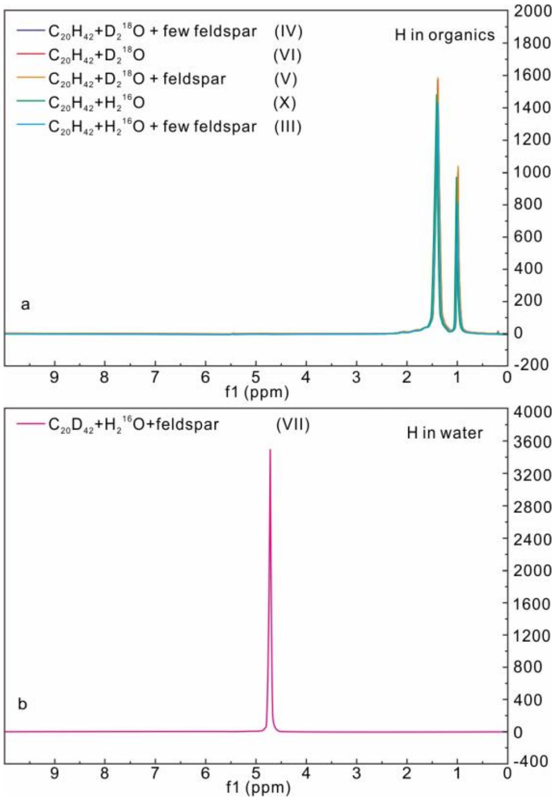

Supplementary Fig. 4. Hydrogen (H)-NMR (nuclear magnetic resonance) of liquid oils and water after thermal experiments. **a** with $C_{20}H_{42}$ serving as the initial alkane samples, strong signals of hydrogen were detected in the post-reaction liquid oils obtained from experiments with and without D-labeled water. **b** with H_2O serving as initial water sample, strong signal of hydrogen was detected in the post-reaction water, even D-labeled $C_{20}D_{42}$ was employed in the experiment. Detailed data have been deposited in Zenodo.

Supplementary Tables

Supplementary Table 1. Compositions of the isotope-tagged thermal experiments conducted in Hastelloy reactors at 340 °C.

No.	*n*-$C_{20}H_{42}$	*n*-$C_{20}D_{42}$	$H_2{}^{16}O$	$D_2{}^{16}O$	$D_2{}^{18}O$	K-feldspar
I	2 g	/	/	/	/	/
II	2 g	/	/	/	/	2 g in mesh bag
III	2 g	/	2 g	/	/	20 mg in mesh bag
IV	2 g	/	/	2 g	/	20 mg in mesh bag
V	2 g	/	/	/	2 g	2 g in mesh bag
VI	2 g	/	/	/	2 g	/
VII	/	2 g	2 g	/	/	2 g in mesh bag
VIII	2 g	/	2 g	/	/	2 g in mesh bag
IX	/	/	2 g	/	/	2 g in mesh bag
X	2 g	/	2 g	/	/	/

Supplementary Table 2. Summary of water and alkane/oil samples used in the in-situ visual thermal experiments.

No.	Compositions		Temperature (°C)	Pressure (MPa)
(1)	Ultrapure water	*n*-$C_{20}H_{42}$	25-410 °C, until complete miscibility	0.1-60
(2)		Liquid hydrocarbons obtained from Experiment-III listed in supplementary Table 1		
(3)		Crude oil from Bohai Bay Basin, East China		

Supplementary Table 3. Isotopic composition of gaseous and liquid alkanes in experiments with (IV) and without (III) D-labeled water.

Alkanes	Experiment-III ($C_{20}H_{42}$ + $H_2{}^{16}O$ + 20mg feldspar)		Experiment-IV ($C_{20}H_{42}$ + $D_2{}^{16}O$ + 20mg feldspar)	
	δD (‰)	AT% D/H	δD (‰)	AT% D/H
CH_4	-287	0.0111	938607	12.7661
C_2H_6	-313	0.0107	185123	2.8172
n-C_9H_{20}	-111	0.0138	17300	0.2842
n-$C_{10}H_{22}$	-162	0.0130	19620	0.3201
n-$C_{11}H_{24}$	-90	0.0142	21451	0.3484
n-$C_{12}H_{26}$	-77	0.0144	22027	0.3574
n-$C_{13}H_{28}$	-69	0.0145	20645	0.3360
n-$C_{14}H_{30}$	-66	0.0145	21488	0.3490
n-$C_{15}H_{32}$	-73	0.0144	16325	0.2691
n-$C_{16}H_{34}$	-67	0.0145	16406	0.2704
n-$C_{17}H_{36}$	-127	0.0136	16287	0.2685
n-$C_{20}H_{42}$	-69	0.0145	5182	0.0962
n-$C_{21}H_{44}$	-33	0.0151	14382	0.2390

Supplementary Table 4. Isotopic composition of CO_2 in experiments with (V, VI) and without (III, IV) ^{18}O-labeled water.

Experiment No.	Species combination	CO_2	
		$\delta^{18}O$ (‰)	AT% $^{18}O/^{16}O$
III	$C_{20}H_{42}$+$H_2{}^{16}O$+ 20mg feldspar	-16.78	0.1967
IV	$C_{20}H_{42}$+$D_2{}^{16}O$+ 20mg feldspar	-16.77	0.1967
V	*n*-$C_{20}H_{42}$ +$D_2{}^{18}O$ +2 g feldspar	60,631	11.00
VI	*n*-$C_{20}H_{42}$ +$D_2{}^{18}O$	95,731	16.25

Supplementary Table 5. Isotopic compositions of the initial water, post-reaction water and clay minerals in experiments with (VII) and without (VIII, IX, X) D-labeled *n*-eicosane.

Experiment No.	Species combination	Water			Clay minerals	
		δD (‰)	AT% D/H	$\delta^{18}O$ (‰)	δD (‰)	AT% D/H
/	Initial water prior to experiment	-31.3	0.01508	-4.97	/	/
VII	*n*-$C_{20}D_{42}$+ H_2O+2g feldspar	36,997	0.5883	-1.17	12,943.22	0.219
VIII	*n*-$C_{20}H_{42}$+ H_2O+2g feldspar	-38.3	0.014953	0.23	162.39	0.018
IX	H_2O+2g feldspar	-36.9	0.014968	-4.08	22.33	0.016
X	*n*-$C_{20}H_{42}$+ H_2O	-37.3	0.014962	-4.73	/	/

Supplementary References

1. Yuan, G. et al. Mixing processes and patterns of fluids in alkane-CO_2-water systems under high temperature and high pressure—Microscopic visual physical thermal simulations and molecular dynamics simulations. *Sci. China Earth Sci.* **66**, 1622-1646 (2023).
