# Supplementary material for: Microdroplets initiate organic-inorganic interactions and mass transfer in thermal hydrous geosystems
Source: Nat Commun. 2024 Jun 11;15:4960. doi: 10.1038/s41467-024-49293-y (PMC11167059; doi:10.1038/s41467-024-49293-y)
Supplement: Supplementary file 3 — Description of Additional Supplementary Files [file 41467_2024_49293_MOESM3_ESM.pdf]

Description of Additional Supplementary Files

Supplementary Movie 1: no formation of water microdroplets in the water-n-C20H42 system at 140 °C

Supplementary Movie 2: formation and evolution of water microdroplets in the water-n-C20H42 system at 150 °C

Supplementary Movie 3: formation and evolution of water microdroplets in the water-n-C20H42 system at 200 °C.

Supplementary Movie 4: formation and evolution of water microdroplets in the water-n-C20H42 system at 300 °C.

Supplementary Movie 5: formation and evolution of water microdroplets in the water-n-C20H42 system at 340 °C

Supplementary Movie 6: no formation of water microdroplets in the water-pyrolized liquid hydrocarbon system at 120 °C

Supplementary Movie 7: formation and evolution of water microdroplets in the water-pyrolized liquid hydrocarbon system at 150 °C.

Supplementary Movie 8: formation and evolution of water microdroplets in the water-pyrolized liquid hydrocarbon system at 280 °C.

Supplementary Movie 9: formation and evolution of hydrocarbon microdroplets in the water-pyrolized liquid hydrocarbon system at 340 °C.

Supplementary Movie 10: no formation of water microdroplets in the water-crude oil system at 120 °C.

Supplementary Movie 11: formation and evolution of water microdroplets in the water-crude oil system at 165 °C.

Supplementary Movie 12: formation and evolution of water microdroplets in the water-crude oil system at 200 °C.

Supplementary Movie 13: formation and evolution of oil microdroplets in the water-crude oil system at 200 °C.

Supplementary Movie 14: formation and evolution of water and oil microdroplets in the water-crude oil system at 250 °C.

Supplementary Movie 15: formation and evolution of water and oil microdroplets in the water-crude oil system at 340 °C.
