# Supplementary material for: Microdroplets initiate organic-inorganic interactions and mass transfer in thermal hydrous geosystems
Source: Nat Commun. 2024 Jun 11;15:4960. doi: 10.1038/s41467-024-49293-y (PMC11167059; doi:10.1038/s41467-024-49293-y)
Supplement: Supplementary file 19 — Reporting Summary [file 41467_2024_49293_MOESM19_ESM.pdf]

nature portfolio

Corresponding author(s): Guanghui Yuan, Yingchang Cao

Last updated by author(s): Apr 28, 2024

Reporting Summary

Nature Portfolio wishes to improve the reproducibility of the work that we publish. This form provides structure for consistency and transparency in reporting. For further information on Nature Portfolio policies, see our Editorial Policies and the Editorial Policy Checklist.

Statistics

For all statistical analyses, confirm that the following items are present in the figure legend, table legend, main text, or Methods section.

n/a	Confirmed	
☒	☐	The exact sample size (*n*) for each experimental group/condition, given as a discrete number and unit of measurement
☐	☒	A statement on whether measurements were taken from distinct samples or whether the same sample was measured repeatedly
☒	☐	The statistical test(s) used AND whether they are one- or two-sided<br>*Only common tests should be described solely by name; describe more complex techniques in the Methods section.*
☒	☐	A description of all covariates tested
☒	☐	A description of any assumptions or corrections, such as tests of normality and adjustment for multiple comparisons
☒	☐	A full description of the statistical parameters including central tendency (e.g. means) or other basic estimates (e.g. regression coefficient) AND variation (e.g. standard deviation) or associated estimates of uncertainty (e.g. confidence intervals)
☒	☐	For null hypothesis testing, the test statistic (e.g. *F*, *t*, *r*) with confidence intervals, effect sizes, degrees of freedom and *P* value noted<br>*Give P values as exact values whenever suitable.*
☒	☐	For Bayesian analysis, information on the choice of priors and Markov chain Monte Carlo settings
☒	☐	For hierarchical and complex designs, identification of the appropriate level for tests and full reporting of outcomes
☒	☐	Estimates of effect sizes (e.g. Cohen's *d*, Pearson's *r*), indicating how they were calculated

Our web collection on statistics for biologists contains articles on many of the points above.

Software and code

Policy information about availability of computer code

Data collection	For collection of GC data, Chemstation c.01.09 in the Agilent 6890N gas chromatograph (GC) system was used; For collection of isotope data, Isodat 3.0 in the Thermo Fisher MAT-253 GC-isotope ratio mass spectrometry (GC-IRMS) system was used; For collection of HF-NMR, Topspin3.5 in the AVANCE III 600MHz HF-NMR spectrometer system was used; For collection of SEM image, NS3.0 in Coxem-30plus scanning electron microscope (SEM) system was used; For collection of Electron paramagnetic resonance (EPR), EPR-ProCt in an EPR spectrometer (CIQTEK EPR200-Plus) system was used. For in-situ observation and recording of microdroplets in silica tubes, Labspec 6.0 and oCam v 430.0 were used.
Data analysis	We used Sigmaplot 11.0, Coredraw 18.0 to prepare the figures.

For manuscripts utilizing custom algorithms or software that are central to the research but not yet described in published literature, software must be made available to editors and reviewers. We strongly encourage code deposition in a community repository (e.g. GitHub). See the Nature Portfolio guidelines for submitting code & software for further information.

Data

Policy information about availability of data

All manuscripts must include a data availability statement. This statement should provide the following information, where applicable:

- Accession codes, unique identifiers, or web links for publicly available datasets
- A description of any restrictions on data availability
- For clinical datasets or third party data, please ensure that the statement adheres to our policy

All data are present in the paper, the supplementary materials, or the files deposited in Figshare.

Research involving human participants, their data, or biological material

Policy information about studies with human participants or human data. See also policy information about sex, gender (identity/presentation), and sexual orientation and race, ethnicity and racism.

Reporting on sex and gender	N/A
Reporting on race, ethnicity, or other socially relevant groupings	N/A
Population characteristics	N/A
Recruitment	N/A
Ethics oversight	N/A

Note that full information on the approval of the study protocol must also be provided in the manuscript.

Field-specific reporting

Please select the one below that is the best fit for your research. If you are not sure, read the appropriate sections before making your selection.

☐ Life sciences ☐ Behavioural & social sciences ☒ Ecological, evolutionary & environmental sciences

For a reference copy of the document with all sections, see nature.com/documents/nr-reporting-summary-flat.pdf

Ecological, evolutionary & environmental sciences study design

All studies must disclose on these points even when the disclosure is negative.

Study description	We conduct isotope-tagged thermal experiments with different combinations of species including water, nC20H42 and feldspar grains and in-situ thermal experiments to study the formation of water microdroplets and relevant impact of organic-inorganic interactions and associated mass transfer in thermal geochemical systems. We obtained gas yields, isotopic composition of gases, liquid hydrocarbons and water from these thermal experiments. The GC analysis were tested for one time, and the device had a relative small error of <0.5%; the isotopic compositions were tested for two to three times to ensure repeatablity, and the analytical uncertainties for the determination of δD and δ18O were better than 2.0‰ and 0.1‰, respectively; the HF-NMR and EPR spectrum were collected at least three times for each samples. Details have been presented in the Methods section.
Research sample	For the thermal experiments in Hastelloy reactors, we employed three compounds—n-C20H42(n-C20D42), H2O (D2O, D218O) and K-feldspar, to explore chemical reactions and H/O transfer across different species . Eicosane, a key petroleum constituent, water, and feldspar are chosen for their relevance in hydrogeochemical reactions and natural prevalence. For the in-situ thermal experiments in silica tubes, we used water and three different types of oils including n-C20H42, the liquid hydrocarbon produced from the pyrolysis of n-C20H42 with H2O, and a deep crude oil from Bohai Bay Basin, East China, to mirror processes in Hastelloy reactors and demonstrate universality of microdroplet generation in both HTHP reactors and deep hydrocarbon reservoirs.
Sampling strategy	We designed ten sets of thermal experiments in Hastellory reactors, with different combinations of n-C20H42(n-C20D42), H2O (D2O, D218O) and K-feldspar. After the thermal experiments, we tested the gas yields, isotopic compositions of gaseous and liquid hydrocarbons, CO2 and water, and HF-NMR of liquid hydrocarbons and water. With comparison of the data from different experiments, we analyzed the occurrence of organic-inorganic interaction and mass transfer. We designed three sets of in-situ thermal experiments to investigate the formation and evolution of microdroplets, with combination of water and three different types of oil.
Data collection	All data were tested by authors G.H.Y., Z.H.J and X.L.H. Some data are tested with the help of technologists, including Dr. Jin Guishan at Analytical Laboratory of BRIUG for the assistance in clay isotope tests; Dr. Liubin Feng at the High-field Nuclear Magnetic Resonance Research Center, Xiamen University for the assistance in HF-NMR tests; Dr. Fang Qing at CIQTEK Co., Ltd. for the assistance in EPR tests.

Timing and spatial scale	The design and conduction of thermal experiments in Hastelloy reactors and relevant testing of gas yield, SEM imgaes, GC of liquid oils, isotopic composition of gases and water, the HF-NMR of water and liquid hydrocarbons were conducted from 2019 to 2022. The isotopic compositions of liquid hydrocarbons were conducted in 2024. The in-situ thermal experiments in silica tubes for testing of microdroplets and the EPR testing of microdroplets were conducted from 2021 to 2024.
Data exclusions	No data were excluded from the analysis.
Reproducibility	The isotopic compositions were tested for two to three times to ensure repeatablity; the HF-NMR and EPR spectrum were collected at least three times for each samples. Also, standards were used to promise the accuracy of the testing data.
Randomization	Randomization is not relevant in this study as all samples were collected from specific thermal experiments and subjected to identical laboratory processing and data collection procedures.
Blinding	Blinding is not relevant in this study. All samples were collected from thermal experiments for specific purposes, and subjected to identical laboratory processing and data collection procedures.

Did the study involve field work? ☐ Yes ☒ No

Reporting for specific materials, systems and methods

We require information from authors about some types of materials, experimental systems and methods used in many studies. Here, indicate whether each material, system or method listed is relevant to your study. If you are not sure if a list item applies to your research, read the appropriate section before selecting a response.

Materials & experimental systems

n/a	Involved in the study
☒	☐ Antibodies
☒	☐ Eukaryotic cell lines
☒	☐ Palaeontology and archaeology
☒	☐ Animals and other organisms
☒	☐ Clinical data
☒	☐ Dual use research of concern
☒	☐ Plants

Methods

n/a	Involved in the study
☒	☐ ChIP-seq
☒	☐ Flow cytometry
☒	☐ MRI-based neuroimaging

Plants

Seed stocks	N/A
Novel plant genotypes	N/A
Authentication	N/A
